# Thesis on Delirium Tremens, or the Madness of Drunkards, &c.

**Published:** 1843-10

**Authors:** 


Art. II.—*Thèse sur le Delirium Tremens, ou Folie des Ivrognes, &c.* Par le Docteur Bougard.—*Bruxelles*, 1843. 8vo, pp. 163.

*Thesis on Delirium Tremens, or the Madness of Drunkards, &c.* By Dr. Bougard.—*Brussels*, 1843.

This thesis was composed on the occasion of the author's obtaining the title of "docteur aggrégé," from the faculty of medicine in the university of Brussels; and like many similar productions has a formal, syllogistic air; and contains not a few needless distinctions and divisions. At the same time, there is to be found in it a considerable amount of practical information and observation. Although we cannot perceive anything essentially novel, in regard either to doctrine or treatment, we can yet, with confidence, recommend the volume as a rather valuable and useful monograph.

